# The complete mitochondrial genome of *Jesogammarus* (*Jesogammarus*) *hinumensis* (Crustacea: Amphipoda: Anisogammaridae)

**DOI:** 10.1080/23802359.2020.1775520

**Published:** 2020-06-12

**Authors:** Chi-Woo Lee, Ko Tomikawa, Gi-Sik Min

**Affiliations:** a Department of Biological Sciences, Inha University, Incheon, Korea; b Department of Science Education, Graduate School of Education, Hiroshima University, Higashihiroshima, Japan

**Keywords:** Complete mitogenome, Amphipoda, *Jesogammarus (Jesogammarus) hinumensis*, brackish water

## Abstract

We determined the mitogenome sequence of *Jesogammarus* (*Jesogammarus*) *hinumensis* Morino, 1993, which is the first complete mitogenome sequence in the family Anisogammaridae Bousfield, 1977. The complete mitogenome of *J*. (*J*.) *hinumensis* was 15,186 bp in length with the typical 13 protein-coding genes (PCGs), 22 transfer RNAs (tRNAs), two ribosomal RNAs (rRNAs), and a control region (CR). The gene order of *J*. (*J*.) *hinumensis* was in accordance with the typical pan-crustacean ground pattern. A maximum-likelihood tree constructed using 25 eumalacostracan mitogenomes confirmed that *J*. (*J*.) *hinumensis* is most closely related to the family Micruropodidae, and supported the monophyly of the superfamily Gammaroidea.

Amphipods are a very important part of many freshwater and marine ecosystems (Barnard and Barnard 1983). Members of the superfamily Gammaroidea are widespread and play important functional roles in fresh and brackish water ecosystems in the northern hemisphere (Bousfield 1979; Jażdżewski 1980). The family Anisogammaridae Bousfield, 1979 is one of the Gammaroidean families endemic to the north Pacific Rim region (Tzvetkova 1975; Bousfield 1979,. The anisogammarid genus *Jesogammarus* Bousfield, 1979 consists of two subgenera (*Annanogammarus* Bousfield, 1979 and *Jesogammarus* Bousfield, 1979), which have been recorded in fresh and brackish waters in the Korean Peninsula, the Japanese archipelago, and the Chinese Continent (Bousfield 1979; Morino 1984; KS Lee and Seo 1990; Hou and Li 2004), and are currently known to include 20 species (Tomikawa et al. 2017). Of these, *Jesogammarus* (*J*.) *hinumensis* Morino, 1993 was originally described from a brackish lake in Japan, named Hinuma, and then on Jeju-island, Korea (Lee et al. 2019).

Previous studies have showed the phylogenetic position of Anisogammaridae within Gammaroidea (Tomikawa et al. 2010; Hou and Sket 2016). Hence, additional knowledge about the mitogenome of the anisogammarid species within Gammaroidea will improve our understanding of the phylogenetic relationships between gammaroidean amphipods.

Individuals of *Jesogammarus* were collected from brackish water in Korea (33°30.19′N, 126°53.51′E). Mitochondrial DNA extraction, sequencing, and gene annotation were performed following the methods described in Song et al. (2016). The extracted mitochondrial DNA is maintained in the DNA collection at the National Institute of Biological Resources, Incheon, South Korea (deposit no. NIBRGR0000620078). A maximum-likelihood tree was constructed using IQ-tree 1.6.3 (Nguyen et al. 2015), based on the concatenated sequences of 10 PCGs (*atp6*, *cox1*, *cox2*, *cox3*, *cytb*, *nad1*, *nad2*, *nad3*, *nad4*, and *nad5*) from 25 eumalacostracan species, including the present sequence and two isopods as outgroup taxa (Figure 1). The *cox1* sequence of the extracted DNA was concordant with NCBI accession numbers LC052235 (Tomikawa 2015) and MN068364 (Lee et al. 2019), thus proving the taxonomic identity of the specimens under study.

**Figure 1.**
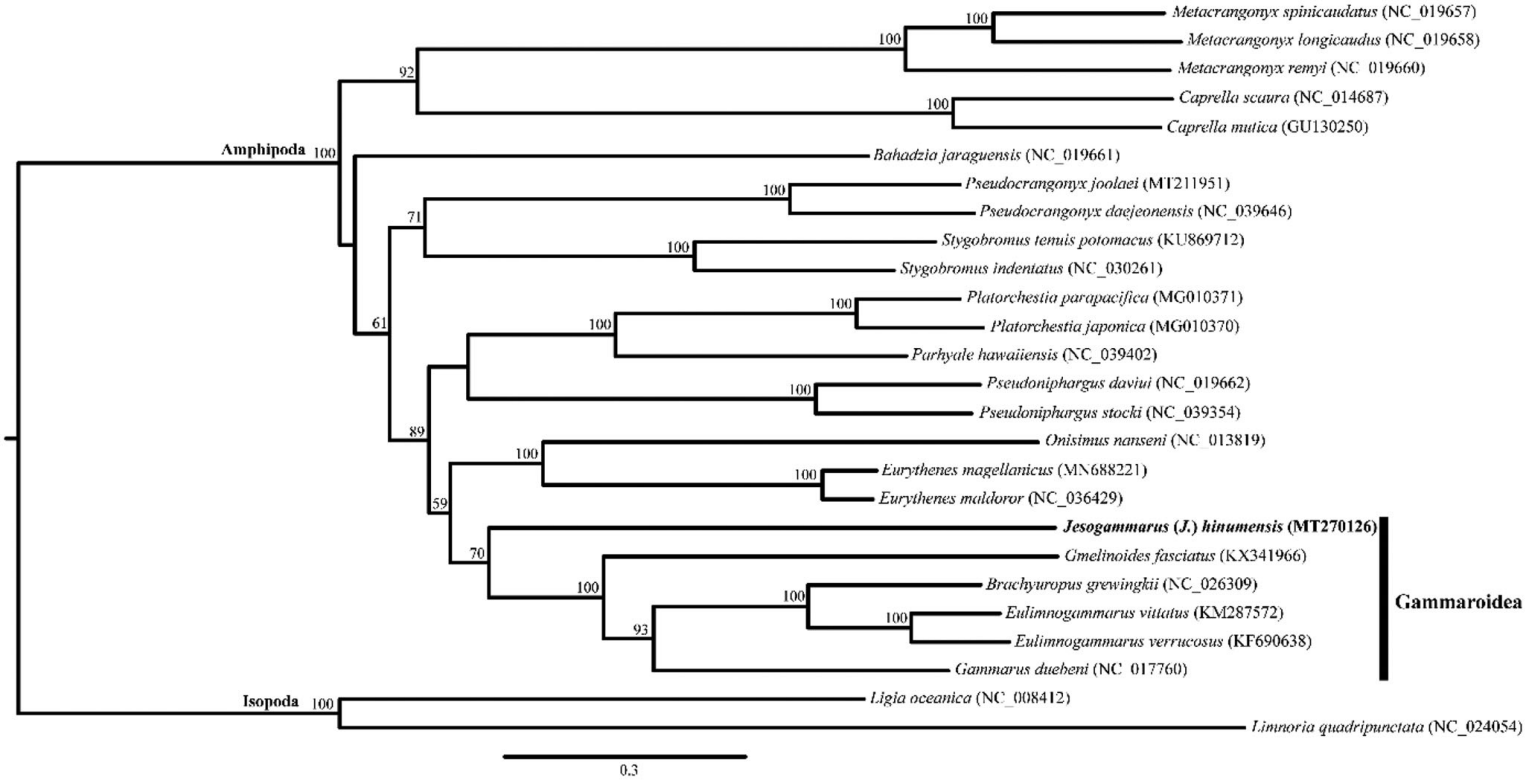
Maximum-likelihood (ML) tree based on the mitogenome sequence of *Jesogammarus* (*Jesogammarus*) *hinumensis* (MT270126) and 25 other eumalacostracan species. The bootstrap supports are shown on each node.

The complete mitogenome of *J*. (*J*.) *hinumensis* (GenBank accession no. MT270126) was 15,186 bp in length and contained the typical 13 PCGs, 22 tRNAs, 2 rRNAs, and a CR. The gene arrangement of *J*. (*J*.) *hinumensis* was concordant with the typical pan-crustacean ground pattern. The obtained maximum-likelihood tree supported the monophyly of the superfamily Gammaroidea, which includes *J*. (*J*.) *hinumensis.*

## Data Availability

The data that support the findings of this study are openly available in GenBank of NCBI at https://www.ncbi.nlm.nih.gov, reference number MT270126.
